# Management of the Patient with an Acute Massive Rise in the Capture Threshold

**Published:** 2001-10-01

**Authors:** Paul A Levine

## Introduction

Since the introduction of steroid eluting electrodes, the incidence of an early massive rise in the capture threshold that either exceeds or threatens to exceed the programmed output of the pacemaker has declined but has not totally disappeared [1]  If a persistent or massive threshold rise is encountered in the days to months post-implant, one consideration is microinstability of the lead.  In this setting, there may be a change in the morphology of the pacemaker evoked depolarization on the ECG or a change in the physical location of the lead as assessed with a chest x-ray.  Another marker is fluctuations in the capture threshold on repeated assessments at the same office or clinic visit. The options for this problem include an operative procedure to reposition or replace the lead or to closely observe the patient hoping that the lead settles into a secure location. Another potential totally reversible cause is the introduction of an new medication or herb. If the possible explanations for threshold increase cited above have been excluded and the high capture threshold is believed to be due to lead maturation, increasing the output or possible lead replacement or repositioning have been the usual options.

A massive capture threshold rise encountered years post-implant is not associated with the acute inflammatory process at the electrode-tissue interface although there is one report in the literature of prednisolone effectively treating a threshold rise occurring two years post-implant [2] The threshold rise in this situation is probably due to either a primary myocardial process resulting in increased fibrosis or scarring at the electrode-tissue interface or a mechanical problem (conductor fracture or damage to the insulation) developing with the lead.   In both of these settings, if an adequate safety margin cannot be maintained by increasing the programmed output, the lead will need to be replaced.

In the mid-1960's, Dr. Preston and colleagues published a number of papers reporting the impact of various pharmacologic agents and physiologic factors on capture thresholds [3,4]. This was further elaborated upon by both these authors [5] and Dr. Sowton [6] at a conference devoted to the emerging science of cardiac pacing.  Glucocorticoids were reported reduce capture thresholds.  As these are also very potent anti-inflammatory agents, it was further postulated that by reducing if not eliminating the acute inflammatory process that accounted for a significant component of the threshold rise during the lead maturation process, one might also use systemic steroids to treat the early massive rise in capture thresholds.  It was these early reports that led to the introduction of steroid (dexamethasone sodium phosphate) into the tip of the modern electrode.   The results have been superb with a marked blunting in the post-implant rise in capture threshold even though this has not been totally eliminated [1].  Despite the routine use of steroid eluting electrodes at this time, the physician will still encounter a rare patient with an unexpected massive rise in the capture threshold.

Over the years, based on an occasional patient whom I am following but more often, at the request of another physician calling to discuss management of a patient with a massive rise in the capture threshold during the lead maturation period (up to 6 months post-implant), I have either tried or recommended a course of oral steroids before proceeding with an operative intervention.  This has been anecdotally successful in approximately 50% of the patients.  If effective, it may eliminate the need for an operative intervention.

I have both used and recommended the following protocol.  It has NOT been subjected to a formal double blind prospective randomized placebo-controlled trial. As such, it must be considered totally empiric. It is unknown whether the "beneficial effect" is actually coincidence having initiated steroids at the peak of the threshold maturation curve which would have started to decline on its own or a true effect.

## Protocol

1. Detailed assessment of the capture threshold.  If the pulse amplitude and pulse width programmability of the pacemaker allow, a full strength duration curve should be obtained.  Also obtain a sensing threshold.  [Note: the acute inflammatory reaction at the electrode-tissue interface will cause a rise in the capture threshold and deterioration in the sensing threshold.]

2. Initiate a trial of Prednisone at 1 mg/kg or 60 mg/day- which ever is lower.  This may be given in divided doses.  This presupposes the absence of a contraindication to systemic steroids.

3. Repeat the capture and sensing threshold assessment in 4 to 5 days.  If there is **NO** change in these thresholds at this evaluation, the steroid is unlikely to be effective and it is recommended that this medication be discontinued.  This is too short a time for adrenal suppression to occur and tapering is not required.

4. If there has been a significant drop in the capture threshold (defined as a decrease in at least two programming steps - either pulse width and/or pulse amplitude), the current dose of prednisone should be continued for a minimum of 1 month.

5. Capture and sensing thresholds should be tracked on a biweekly basis after demonstrating the initial improvement.

6. At the end of 1 month on the relatively high prednisone dose, begin to slowly taper the steroids planning to discontinue them after a 2 month course of slow tapering.

7. During this time, it is important to closely monitor capture and sensing threshold. If these are demonstrated to deteriorate, the prednisone dose should be increased for a couple of weeks and tapering only resumed after the thresholds are shown to again improve.

8. If the steroid is initially ineffective or effective but the threshold does not fall to a sufficiently low level such that lead repositioning is planned, the likelihood of a high threshold occurring with the second procedure is in the range of 4 to 5%.  My recommendation would be to use a new lead rather than trying to reposition the current lead. If a steroid-eluting lead had not been used on the first procedure, one should be used at this time.  If a steroid-eluting lead had been used on the initial procedure, a new lead should still be used as traction on the original lead to disengage it from the primary position may disrupt the mechanical integrity of the lead predisposing to late problems.  Damage to the lead may not be immediately apparent upon testing with a Pacing System Analyzer.  In both cases, I recommend the use of a steroid-eluting active fixation lead so that the electrode can be positioned in an area remote from the original location.

9. If the decision is made to proceed with a repeat operative procedure involving either replacement or repositioning of the lead, a venogram is strongly recommended.  This should be performed just prior to the procedure to determine if the central venous system is patent and will allow repeat access for placement of a new lead.

## Caution

Possible side effects that are associated with a protracted course of high dose steroid therapy include but are not limited to suppression of the adrenal and pituitary glands, impairment of immunocompetence and thus predisposition to infections, impairment of wound healing, exacerbation of peptic ulcer disease, acceleration of osteoporosis, induction of fluid retention and electrolyte imbalance, and exacerbation of diabetes mellitus.  The Physician's Desk Reference or a major pharmacology text should be checked if one is not sure of all the potential complications associated with systemic steroid therapy.  The patient should be both evaluated and followed for the potential complications associated with systemic steroids during the course of this treatment.  If a complication develops, it will be a clinical decision to either treat the complications and continue the steroids or to discontinue the steroids.  The physician must determine whether the potential benefits to be gained from the use of systemic steroids outweigh the theoretical risks vs the potential for loss of capture and need for a repeat operation.
